# 6-(4-Amino­phen­yl)-4-(4-eth­oxy­phen­yl)-2-meth­oxy­nicotinonitrile

**DOI:** 10.1107/S1600536812036276

**Published:** 2012-08-31

**Authors:** Thitipone Suwunwong, Suchada Chantrapromma, Hoong-Kun Fun

**Affiliations:** a Department of Chemistry and Center of Excellence for Innovation in Chemistry, Faculty of Science, Prince of Songkla University, Hat-Yai, Songkhla 90112, Thailand; b Crystal Materials Research Unit, Department of Chemistry, Faculty of Science, Prince of Songkla University, Hat-Yai, Songkhla 90112, Thailand; c X-ray Crystallography Unit, School of Physics, Universiti Sains Malaysia, 11800 USM, Penang, Malaysia

## Abstract

In the title mol­ecule, C_21_H_19_N_3_O_2_, the central pyridine ring makes dihedral angles of 14.46 (9) and 34.67 (8)° with the 4-amino- and 4-eth­oxy-substituted benzene rings, respectively. The eth­oxy group is essentially coplanar with the attached benzene ring [C—O—C—C torsion angle = 178.70 (16)°] as is the meth­oxy group with the pyridine ring [C—O—C—N torsion angle = −3.0 (3)°]. In the crystal, mol­ecules are linked by N—H⋯N hydrogen bonds into chains along [201]. Weak C—H⋯O hydrogen bonds and C—H⋯π inter­actions are also present.

## Related literature

The title nicotinonitrile derivative is a cyclized product of a chalcone and a malononitrile in the present of sodium methoxide. For the synthesis and applications of substituted pyridines and nicotinonitrile derivatives, see: Al-Jaber *et al.* (2012 ▶); Brandt *et al.* (2010 ▶); El-Sayed *et al.* (2011 ▶); Goda *et al.* (2004 ▶); Ji *et al.* (2007 ▶); Kamal *et al.* (2007 ▶); Kim *et al.* (2005 ▶); Kolev *et al.* (2005 ▶); Koner *et al.* (2012 ▶); Zhou *et al.* (2006 ▶). For a related structure, see: Chantrapromma *et al.* (2010 ▶). For standard bond-length data, see: Allen *et al.* (1987 ▶).
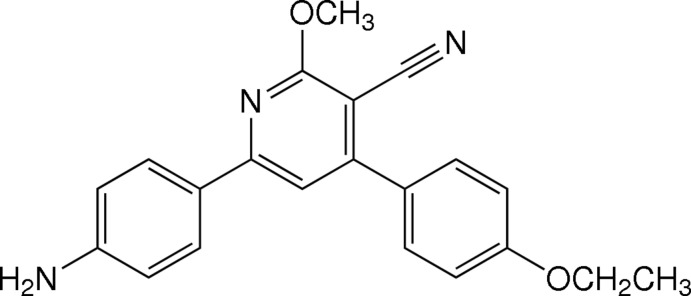


## Experimental

### 

#### Crystal data

C_21_H_19_N_3_O_2_
*M* *_r_* = 345.39
Monoclinic, 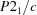

*a* = 5.3924 (2) Å
*b* = 16.5111 (5) Å
*c* = 20.1415 (6) Å
β = 91.315 (2)°
*V* = 1792.82 (10) Å^3^
*Z* = 4
Mo *K*α radiation
μ = 0.08 mm^−1^
*T* = 298 K
0.54 × 0.25 × 0.22 mm

#### Data collection

Bruker APEXII CCD area-detector diffractometer
Absorption correction: multi-scan (*SADABS*; Bruker, 2005 ▶) *T*_min_ = 0.956, *T*_max_ = 0.982
17814 measured reflections
5216 independent reflections
3013 reflections with *I* > 2σ(*I*)
*R*_int_ = 0.028

#### Refinement

*R*[*F* ^2^ > 2σ(*F* ^2^)] = 0.052
*wR*(*F* ^2^) = 0.159
*S* = 1.04
5216 reflections
245 parameters
H atoms treated by a mixture of independent and constrained refinement
Δρ_max_ = 0.19 e Å^−3^
Δρ_min_ = −0.17 e Å^−3^

### 

Data collection: *APEX2* (Bruker, 2005 ▶); cell refinement: *SAINT* (Bruker, 2005 ▶); data reduction: *SAINT*; program(s) used to solve structure: *SHELXTL* (Sheldrick, 2008 ▶); program(s) used to refine structure: *SHELXTL*; molecular graphics: *SHELXTL*; software used to prepare material for publication: *SHELXTL* and *PLATON* (Spek, 2009 ▶).

## Supplementary Material

Crystal structure: contains datablock(s) global, I. DOI: 10.1107/S1600536812036276/lh5514sup1.cif

Structure factors: contains datablock(s) I. DOI: 10.1107/S1600536812036276/lh5514Isup2.hkl

Supplementary material file. DOI: 10.1107/S1600536812036276/lh5514Isup3.cml

Additional supplementary materials:  crystallographic information; 3D view; checkCIF report

## Figures and Tables

**Table 1:** Hydrogen-bond geometry (Å, °) *Cg* is the centroid of the C12–C17 ring.

*D*—H⋯*A*	*D*—H	H⋯*A*	*D*⋯*A*	*D*—H⋯*A*
N3—H2*N*3⋯N2^i^	0.88 (3)	2.20 (3)	3.084 (3)	177 (2)
C21—H21*A*⋯O1^ii^	0.96	2.52	3.439 (2)	160
C18—H18*A*⋯*Cg* ^iii^	0.96	2.84	3.7135 (18)	151

Symmetry codes: (i) 
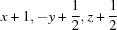
; (ii) 
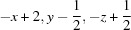
; (iii) 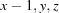.

**Fig. 1.**
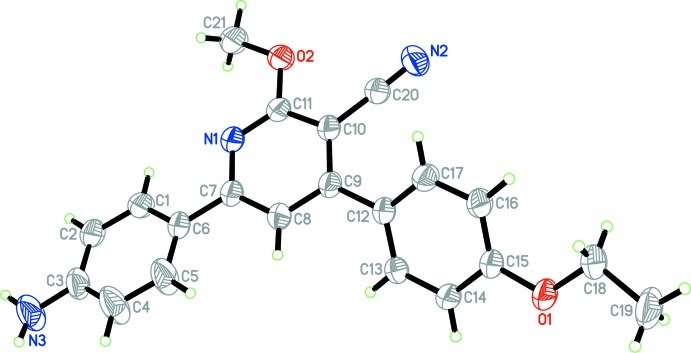
The molecular structure of the title compound, showing 50% probability displacement ellipsoids.

**Fig. 2.**
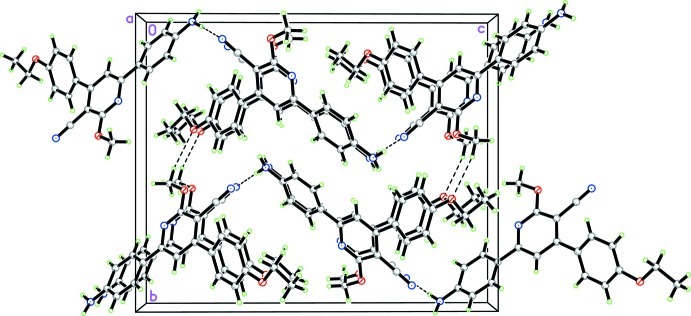
The crystal packing of the title compound viewed along the a axis. Hydrogen bonds are shown as dashed lines.

## supplementary crystallographic information

### Comment

Pyridines have been reported for their applications in a number of areas (Goda *et al.*, 2004; Kamal *et al.*, 2007; Kolev *et al.*, 2005). There are several methods reported for the synthesis of substituted pyridine derivatives including nicotinonitrile derivatives (Al-Jaber *et al.*, 2012; Zhou *et al.*, 2006). Nicotinonitrile derivatives have a wide range of applications such as antitumor, antimicrobial, analgesic, anti-hyperglycemic and antiproliferative activities (Brandt *et al.*, 2010; El-Sayed *et al.*, 2011; Ji *et al.*, 2007; Kim *et al.*, 2005) and fluorescent materials (Koner *et al.*, 2012). Our research is aimed at the synthesis and preliminary fluorescent and antibacterial screening of nicotinonitrile derivatives. The title compound (I) was synthesized by the cyclization of a chalcone derivative with malononitrile to investigate its fluorescent properties. It was found that (I) exhibits fluorescence with the maximum emission at 498 nm when was excited at 370 nm in DMSO.

The molecular structure of the title compound is shown in Fig. 1. The central pyridine ring is inclined to the 4-aminophenyl and 4-ethoxyphenyl rings with the dihedral angles of 14.46 (9) and 34.67 (8)°, respectively. The dihedral angle between these two substituted benzene rings is 44.84 (9)°. The ethoxy substituent of the 4-ethoxyphenyl group is essentially co-planar with the attached benzene ring with the torsion angle C15–O1–C18–C19 = 178.70 (16)° and C18–O1–C15–C16 = 1.8 (3)°. The methoxy group is also approximately co-planar to the pyridine ring as indictated by the torsion angle C21–O2–C11–N1 = -3.0 (3)°. The bond distances agree with the literature values (Allen *et al.*, 1987) and are comparable with those for a related structure (Chantrapromma *et al.*, 2010).

In the crystal (Fig. 2), molecules are linked by N—H···N hydrogen bonds into chains along [201]. Weak C—H···O hydrogen bonds and C—H···π interactions are also present (Table 1).

### Experimental

The title compound (I) was synthesized by stirring the solution of (*E*)-1-(4-aminophenyl)-3-(4-ethoxyphenyl)prop-2-en-1-one (0.27 g, 1 mmol) in methanol (10 ml) with a freshly prepared sodium methoxide (1.0 mmol of sodium in 20 ml of methanol). Excess malononitrile (0.13 g, 2.0 mmol) was then added with continuous stirring at room temperature until the precipitate was separated out. The resulting solid was filtered. Pale brown block-shaped single crystals of the title compound suitable for X-ray structure determination were recrystallized from methanol/ethanol (1:1 *v*/*v*) by the slow evaporation of the solvent at room temperature over several days, Mp. 477–478 K.

### Refinement

Amino H atoms were located in difference maps and refined isotropically. The remaining H atoms were positioned geometrically and allowed to ride on their parent atoms, with d(C—H) = 0.93 Å for aromatic, 0.97 for CH_2_ and 0.96 Å for CH_3_ atoms. The *U*_iso_ values were constrained to be 1.5*U*_eq_ of the carrier atom for methyl H atoms and 1.2*U*_eq_ for the remaining H atoms. A rotating group model was used for the methyl groups.

### Crystal data

**Table:**

C_21_H_19_N_3_O_2_	*F*(000) = 728
*M**_r_* = 345.39	*D*_x_ = 1.280 Mg m^−^^3^
Monoclinic, *P*2_1_/*c*	Melting point = 477–478 K
Hall symbol: -P 2ybc	Mo *K*α radiation, λ = 0.71073 Å
*a* = 5.3924 (2) Å	Cell parameters from 5216 reflections
*b* = 16.5111 (5) Å	θ = 2.0–30.0°
*c* = 20.1415 (6) Å	µ = 0.08 mm^−^^1^
β = 91.315 (2)°	*T* = 298 K
*V* = 1792.82 (10) Å^3^	Block, pale-brown
*Z* = 4	0.54 × 0.25 × 0.22 mm

### Data collection

**Table:**

Bruker APEXII CCD area-detector diffractometer	5216 independent reflections
Radiation source: sealed tube	3013 reflections with *I* > 2σ(*I*)
Graphite monochromator	*R*_int_ = 0.028
φ and ω scans	θ_max_ = 30.0°, θ_min_ = 2.0°
Absorption correction: multi-scan (*SADABS*; Bruker, 2005)	*h* = −7→7
*T*_min_ = 0.956, *T*_max_ = 0.982	*k* = −23→23
17814 measured reflections	*l* = −28→28

### Refinement

**Table:**

Refinement on *F*^2^	Primary atom site location: structure-invariant direct methods
Least-squares matrix: full	Secondary atom site location: difference Fourier map
*R*[*F*^2^ > 2σ(*F*^2^)] = 0.052	Hydrogen site location: inferred from neighbouring sites
*wR*(*F*^2^) = 0.159	H atoms treated by a mixture of independent and constrained refinement
*S* = 1.04	*w* = 1/[σ^2^(*F*_o_^2^) + (0.0647*P*)^2^ + 0.3033*P*] where *P* = (*F*_o_^2^ + 2*F*_c_^2^)/3
5216 reflections	(Δ/σ)_max_ = 0.001
245 parameters	Δρ_max_ = 0.19 e Å^−^^3^
0 restraints	Δρ_min_ = −0.17 e Å^−^^3^

### Special details

**Table:**

Geometry. All e.s.d.'s (except the e.s.d. in the dihedral angle between two l.s. planes) are estimated using the full covariance matrix. The cell e.s.d.'s are taken into account individually in the estimation of e.s.d.'s in distances, angles and torsion angles; correlations between e.s.d.'s in cell parameters are only used when they are defined by crystal symmetry. An approximate (isotropic) treatment of cell e.s.d.'s is used for estimating e.s.d.'s involving l.s. planes.
Refinement. Refinement of *F*^2^ against ALL reflections. The weighted *R*-factor *wR* and goodness of fit *S* are based on *F*^2^, conventional *R*-factors *R* are based on *F*, with *F* set to zero for negative *F*^2^. The threshold expression of *F*^2^ > σ(*F*^2^) is used only for calculating *R*-factors(gt) *etc*. and is not relevant to the choice of reflections for refinement. *R*-factors based on *F*^2^ are statistically about twice as large as those based on *F*, and *R*- factors based on ALL data will be even larger.

### Fractional atomic coordinates and isotropic or equivalent isotropic displacement parameters (Å^2^)

**Table:**

	*x*	*y*	*z*	*U*_iso_*/*U*_eq_	
O1	0.3134 (2)	0.38320 (7)	0.14470 (6)	0.0637 (3)	
O2	1.3927 (3)	0.09389 (7)	0.37553 (7)	0.0737 (4)	
N1	1.3663 (3)	0.21531 (8)	0.43089 (6)	0.0543 (3)	
N2	1.0455 (4)	0.08668 (10)	0.23879 (9)	0.0858 (6)	
N3	1.6313 (5)	0.48378 (15)	0.64781 (10)	0.0987 (7)	
C1	1.5687 (4)	0.32043 (10)	0.52550 (9)	0.0654 (5)	
H1A	1.6556	0.2743	0.5133	0.078*	
C2	1.6575 (4)	0.36652 (11)	0.57804 (9)	0.0675 (5)	
H2A	1.8018	0.3506	0.6006	0.081*	
C3	1.5363 (4)	0.43554 (12)	0.59760 (9)	0.0663 (5)	
C4	1.3212 (5)	0.45547 (16)	0.56331 (12)	0.1042 (9)	
H4A	1.2332	0.5012	0.5759	0.125*	
C5	1.2329 (4)	0.40959 (14)	0.51089 (11)	0.0874 (7)	
H5A	1.0877	0.4254	0.4887	0.105*	
C6	1.3538 (3)	0.34094 (10)	0.49045 (8)	0.0514 (4)	
C7	1.2622 (3)	0.28980 (9)	0.43520 (8)	0.0501 (4)	
C8	1.0805 (3)	0.31513 (10)	0.38963 (8)	0.0540 (4)	
H8A	1.0110	0.3663	0.3941	0.065*	
C9	1.0004 (3)	0.26572 (9)	0.33750 (7)	0.0485 (4)	
C10	1.1104 (3)	0.18863 (9)	0.33383 (8)	0.0509 (4)	
C11	1.2909 (3)	0.16807 (9)	0.38214 (8)	0.0537 (4)	
C12	0.8139 (3)	0.29470 (9)	0.28753 (7)	0.0484 (4)	
C13	0.8087 (3)	0.37611 (10)	0.26827 (8)	0.0547 (4)	
H13A	0.9214	0.4122	0.2876	0.066*	
C14	0.6399 (3)	0.40372 (10)	0.22128 (8)	0.0564 (4)	
H14A	0.6392	0.4581	0.2093	0.068*	
C15	0.4699 (3)	0.35068 (10)	0.19151 (8)	0.0511 (4)	
C16	0.4713 (3)	0.27011 (10)	0.21019 (8)	0.0544 (4)	
H16A	0.3585	0.2341	0.1908	0.065*	
C17	0.6410 (3)	0.24309 (10)	0.25784 (8)	0.0534 (4)	
H17A	0.6391	0.1889	0.2703	0.064*	
C18	0.1324 (3)	0.33169 (12)	0.11351 (9)	0.0643 (5)	
H18A	0.0257	0.3082	0.1465	0.077*	
H18B	0.2131	0.2881	0.0900	0.077*	
C19	−0.0167 (4)	0.38315 (14)	0.06586 (11)	0.0864 (7)	
H19A	−0.1479	0.3513	0.0461	0.130*	
H19B	0.0887	0.4030	0.0317	0.130*	
H19C	−0.0865	0.4281	0.0893	0.130*	
C20	1.0686 (3)	0.13264 (10)	0.28075 (9)	0.0608 (5)	
C21	1.5900 (4)	0.07236 (12)	0.42137 (11)	0.0818 (7)	
H21A	1.6516	0.0196	0.4105	0.123*	
H21B	1.5285	0.0719	0.4657	0.123*	
H21C	1.7217	0.1112	0.4185	0.123*	
H2N3	1.747 (5)	0.4641 (15)	0.6750 (13)	0.101 (8)*	
H1N3	1.536 (5)	0.5205 (19)	0.6634 (15)	0.129 (11)*	

### Atomic displacement parameters (Å^2^)

**Table:**

	*U*^11^	*U*^22^	*U*^33^	*U*^12^	*U*^13^	*U*^23^
O1	0.0660 (7)	0.0607 (7)	0.0629 (8)	−0.0029 (6)	−0.0309 (6)	0.0055 (6)
O2	0.0944 (9)	0.0481 (6)	0.0765 (9)	0.0129 (6)	−0.0432 (8)	−0.0048 (6)
N1	0.0654 (8)	0.0489 (7)	0.0478 (7)	−0.0002 (6)	−0.0171 (6)	0.0033 (6)
N2	0.1065 (14)	0.0644 (10)	0.0843 (12)	0.0134 (9)	−0.0443 (11)	−0.0191 (9)
N3	0.1089 (16)	0.1055 (16)	0.0794 (13)	0.0313 (13)	−0.0496 (12)	−0.0399 (12)
C1	0.0775 (12)	0.0515 (9)	0.0655 (11)	0.0122 (8)	−0.0303 (9)	−0.0019 (8)
C2	0.0783 (12)	0.0623 (10)	0.0603 (11)	0.0073 (9)	−0.0347 (9)	0.0019 (8)
C3	0.0720 (11)	0.0775 (12)	0.0485 (10)	0.0090 (9)	−0.0192 (9)	−0.0112 (8)
C4	0.0993 (16)	0.1191 (19)	0.0918 (16)	0.0551 (14)	−0.0506 (13)	−0.0563 (15)
C5	0.0796 (13)	0.1055 (16)	0.0750 (14)	0.0393 (12)	−0.0402 (11)	−0.0366 (12)
C6	0.0576 (9)	0.0548 (9)	0.0411 (8)	0.0016 (7)	−0.0128 (7)	0.0017 (7)
C7	0.0555 (9)	0.0513 (8)	0.0431 (8)	−0.0004 (7)	−0.0106 (7)	0.0030 (6)
C8	0.0626 (10)	0.0513 (8)	0.0472 (9)	0.0068 (7)	−0.0148 (7)	−0.0027 (7)
C9	0.0510 (8)	0.0499 (8)	0.0439 (8)	−0.0027 (7)	−0.0108 (7)	0.0037 (6)
C10	0.0586 (9)	0.0466 (8)	0.0468 (8)	−0.0045 (7)	−0.0156 (7)	0.0023 (6)
C11	0.0651 (10)	0.0436 (8)	0.0516 (9)	0.0004 (7)	−0.0162 (8)	0.0048 (7)
C12	0.0505 (8)	0.0499 (8)	0.0442 (8)	0.0006 (7)	−0.0123 (7)	−0.0004 (6)
C13	0.0628 (10)	0.0474 (8)	0.0530 (9)	−0.0030 (7)	−0.0209 (8)	−0.0033 (7)
C14	0.0675 (10)	0.0444 (8)	0.0562 (10)	0.0000 (7)	−0.0212 (8)	0.0020 (7)
C15	0.0523 (8)	0.0531 (8)	0.0471 (9)	0.0016 (7)	−0.0146 (7)	0.0007 (7)
C16	0.0503 (9)	0.0545 (9)	0.0576 (10)	−0.0071 (7)	−0.0172 (7)	0.0012 (7)
C17	0.0543 (9)	0.0487 (8)	0.0568 (9)	−0.0036 (7)	−0.0122 (7)	0.0068 (7)
C18	0.0598 (10)	0.0711 (11)	0.0608 (11)	−0.0038 (8)	−0.0226 (8)	−0.0032 (8)
C19	0.0809 (14)	0.0989 (16)	0.0775 (14)	−0.0139 (12)	−0.0418 (11)	0.0149 (12)
C20	0.0703 (11)	0.0479 (8)	0.0630 (11)	0.0020 (8)	−0.0256 (9)	−0.0007 (8)
C21	0.0986 (15)	0.0603 (11)	0.0843 (14)	0.0193 (10)	−0.0444 (12)	−0.0005 (10)

### Geometric parameters (Å, º)

**Table:**

O1—C15	1.3612 (17)	C9—C10	1.407 (2)
O1—C18	1.4291 (19)	C9—C12	1.485 (2)
O2—C11	1.3502 (19)	C10—C11	1.402 (2)
O2—C21	1.437 (2)	C10—C20	1.427 (2)
N1—C11	1.3114 (19)	C12—C17	1.388 (2)
N1—C7	1.356 (2)	C12—C13	1.399 (2)
N2—C20	1.141 (2)	C13—C14	1.376 (2)
N3—C3	1.377 (2)	C13—H13A	0.9300
N3—H2N3	0.88 (3)	C14—C15	1.393 (2)
N3—H1N3	0.86 (3)	C14—H14A	0.9300
C1—C2	1.380 (2)	C15—C16	1.382 (2)
C1—C6	1.385 (2)	C16—C17	1.385 (2)
C1—H1A	0.9300	C16—H16A	0.9300
C2—C3	1.376 (3)	C17—H17A	0.9300
C2—H2A	0.9300	C18—C19	1.501 (3)
C3—C4	1.376 (3)	C18—H18A	0.9700
C4—C5	1.375 (3)	C18—H18B	0.9700
C4—H4A	0.9300	C19—H19A	0.9600
C5—C6	1.375 (3)	C19—H19B	0.9600
C5—H5A	0.9300	C19—H19C	0.9600
C6—C7	1.473 (2)	C21—H21A	0.9600
C7—C8	1.391 (2)	C21—H21B	0.9600
C8—C9	1.391 (2)	C21—H21C	0.9600
C8—H8A	0.9300		
			
C15—O1—C18	118.42 (13)	O2—C11—C10	115.38 (14)
C11—O2—C21	117.24 (13)	C17—C12—C13	117.46 (14)
C11—N1—C7	117.77 (13)	C17—C12—C9	122.12 (14)
C3—N3—H2N3	119.3 (17)	C13—C12—C9	120.42 (13)
C3—N3—H1N3	117 (2)	C14—C13—C12	121.20 (14)
H2N3—N3—H1N3	117 (3)	C14—C13—H13A	119.4
C2—C1—C6	121.82 (17)	C12—C13—H13A	119.4
C2—C1—H1A	119.1	C13—C14—C15	120.40 (15)
C6—C1—H1A	119.1	C13—C14—H14A	119.8
C3—C2—C1	121.18 (16)	C15—C14—H14A	119.8
C3—C2—H2A	119.4	O1—C15—C16	124.59 (14)
C1—C2—H2A	119.4	O1—C15—C14	116.19 (14)
C2—C3—C4	117.04 (17)	C16—C15—C14	119.22 (14)
C2—C3—N3	121.22 (18)	C15—C16—C17	119.88 (14)
C4—C3—N3	121.7 (2)	C15—C16—H16A	120.1
C5—C4—C3	121.77 (19)	C17—C16—H16A	120.1
C5—C4—H4A	119.1	C16—C17—C12	121.83 (15)
C3—C4—H4A	119.1	C16—C17—H17A	119.1
C6—C5—C4	121.70 (17)	C12—C17—H17A	119.1
C6—C5—H5A	119.1	O1—C18—C19	107.14 (15)
C4—C5—H5A	119.1	O1—C18—H18A	110.3
C5—C6—C1	116.47 (15)	C19—C18—H18A	110.3
C5—C6—C7	123.06 (15)	O1—C18—H18B	110.3
C1—C6—C7	120.46 (15)	C19—C18—H18B	110.3
N1—C7—C8	121.12 (14)	H18A—C18—H18B	108.5
N1—C7—C6	115.86 (13)	C18—C19—H19A	109.5
C8—C7—C6	123.02 (14)	C18—C19—H19B	109.5
C9—C8—C7	121.57 (15)	H19A—C19—H19B	109.5
C9—C8—H8A	119.2	C18—C19—H19C	109.5
C7—C8—H8A	119.2	H19A—C19—H19C	109.5
C8—C9—C10	116.51 (13)	H19B—C19—H19C	109.5
C8—C9—C12	121.09 (14)	N2—C20—C10	177.0 (2)
C10—C9—C12	122.38 (13)	O2—C21—H21A	109.5
C11—C10—C9	118.00 (14)	O2—C21—H21B	109.5
C11—C10—C20	117.27 (14)	H21A—C21—H21B	109.5
C9—C10—C20	124.46 (13)	O2—C21—H21C	109.5
N1—C11—O2	119.58 (13)	H21A—C21—H21C	109.5
N1—C11—C10	125.03 (15)	H21B—C21—H21C	109.5
			
C6—C1—C2—C3	−0.5 (3)	C7—N1—C11—C10	0.1 (3)
C1—C2—C3—C4	1.2 (3)	C21—O2—C11—N1	−3.0 (3)
C1—C2—C3—N3	−176.7 (2)	C21—O2—C11—C10	176.03 (17)
C2—C3—C4—C5	−1.3 (4)	C9—C10—C11—N1	0.0 (3)
N3—C3—C4—C5	176.6 (3)	C20—C10—C11—N1	174.15 (17)
C3—C4—C5—C6	0.7 (5)	C9—C10—C11—O2	−179.00 (15)
C4—C5—C6—C1	0.1 (4)	C20—C10—C11—O2	−4.8 (2)
C4—C5—C6—C7	179.0 (2)	C8—C9—C12—C17	−146.49 (17)
C2—C1—C6—C5	−0.2 (3)	C10—C9—C12—C17	35.4 (2)
C2—C1—C6—C7	−179.14 (17)	C8—C9—C12—C13	34.1 (2)
C11—N1—C7—C8	0.3 (2)	C10—C9—C12—C13	−144.01 (17)
C11—N1—C7—C6	−179.60 (15)	C17—C12—C13—C14	−0.5 (3)
C5—C6—C7—N1	−165.10 (19)	C9—C12—C13—C14	178.94 (16)
C1—C6—C7—N1	13.8 (2)	C12—C13—C14—C15	−0.2 (3)
C5—C6—C7—C8	15.0 (3)	C18—O1—C15—C16	1.8 (3)
C1—C6—C7—C8	−166.12 (17)	C18—O1—C15—C14	−178.89 (15)
N1—C7—C8—C9	−0.8 (3)	C13—C14—C15—O1	−178.79 (16)
C6—C7—C8—C9	179.16 (16)	C13—C14—C15—C16	0.6 (3)
C7—C8—C9—C10	0.8 (2)	O1—C15—C16—C17	179.15 (16)
C7—C8—C9—C12	−177.43 (15)	C14—C15—C16—C17	−0.2 (3)
C8—C9—C10—C11	−0.4 (2)	C15—C16—C17—C12	−0.6 (3)
C12—C9—C10—C11	177.79 (15)	C13—C12—C17—C16	1.0 (3)
C8—C9—C10—C20	−174.09 (17)	C9—C12—C17—C16	−178.51 (16)
C12—C9—C10—C20	4.1 (3)	C15—O1—C18—C19	178.70 (16)
C7—N1—C11—O2	178.99 (16)		

### Hydrogen-bond geometry (Å, º)

*Cg* is the centroid of the C12–C17 ring.

**Table:**

*D*—H···*A*	*D*—H	H···*A*	*D*···*A*	*D*—H···*A*
N3—H2*N*3···N2^i^	0.88 (3)	2.20 (3)	3.084 (3)	177 (2)
C21—H21*A*···O1^ii^	0.96	2.52	3.439 (2)	160
C18—H18*A*···*Cg*^iii^	0.96	2.84	3.7135 (18)	151

Symmetry codes: (i) *x*+1, −*y*+1/2, *z*+1/2; (ii) −*x*+2, *y*−1/2, −*z*+1/2; (iii) *x*−1, *y*, *z*.
